# Study of zeolite influence on analytical characteristics of urea biosensor based on ion-selective field-effect transistors

**DOI:** 10.1186/1556-276X-9-124

**Published:** 2014-03-17

**Authors:** Margaryta K Shelyakina, Oleksandr O Soldatkin, Valentyna M Arkhypova, Berna O Kasap, Burcu Akata, Sergei V Dzyadevych

**Affiliations:** 1 Laboratory of Biomolecular Electronics, Institute of Molecular Biology and Genetics of National Academy of Sciences of Ukraine, 150 Zabolotnogo St., Kyiv 03680, Ukraine; 2 Institute of High Technologies, Taras Shevchenko National University of Kyiv, 64 Volodymyrska St., Kyiv 01003, Ukraine; 3 Micro and Nanotechnology Department, Middle East Technical University, Ankara 06531, Turkey; 4 Central Laboratory, Middle East Technical University, Ankara 06531, Turkey; 5 Max Planck Institute for Polymer Research, Ackermannweg 10, Mainz 55128, Germany

**Keywords:** Biosensor, Urease, Silicalite, Zeolite, Nano beta, Nano L, pH-sensitive field-effect transistor

## Abstract

A possibility of the creation of potentiometric biosensor by adsorption of enzyme urease on zeolite was investigated. Several variants of zeolites (nano beta, calcinated nano beta, silicalite, and nano L) were chosen for experiments. The surface of pH-sensitive field-effect transistors was modified with particles of zeolites, and then the enzyme was adsorbed. As a control, we used the method of enzyme immobilization in glutaraldehyde vapour (without zeolites). It was shown that all used zeolites can serve as adsorbents (with different effectiveness). The biosensors obtained by urease adsorption on zeolites were characterized by good analytical parameters (signal reproducibility, linear range, detection limit and the minimal drift factor of a baseline). In this work, it was shown that modification of the surface of pH-sensitive field-effect transistors with zeolites can improve some characteristics of biosensors.

## Background

Zeolites are microporous crystalline solids with well-defined structures. Generally, they contain silicon, aluminium and oxygen in their framework and cations, water and/or other molecules within their pores. Many of them occur naturally as minerals and are extensively mined in different parts of the world. Others are synthetic, and are made commercially for specific applications, or produced by research scientists trying to understand more about their chemistry [1].

A defining feature of zeolites is that their frameworks are made up of four connected networks of atoms. One can think about this in terms of tetrahedral, with a silicon atom in the middle and oxygen atoms at the corners. These tetrahedra can then link together by their corners to form a rich variety of beautiful structures. The framework structure may contain linked cages, cavities or channels, which are of the proper size to allow small molecules to enter [2].

The shape-selective properties of zeolites are also the basis for their use in molecular adsorption. The ability to adsorb preferentially certain molecules, while excluding others, has opened up a wide range of molecular sieving applications. Sometimes, it is simply a matter of the size and shape of pores controlling access into the zeolite. In other cases, different types of molecule enter the zeolite, but some diffuse through the channels more quickly, leaving others stuck behind, as in the purification of para-xylene by silicalite [3].

Cation-containing zeolites are extensively used as desiccants due to their high affinity for water, and also find application in gas separation, where molecules are differentiated on the basis of their electrostatic interactions with the metal ions. Conversely, hydrophobic silica zeolites preferentially absorb organic solvents. Zeolites can thus separate molecules based on differences of size, shape and polarity.

Nowadays, zeolites are of great interest due to their high surface areas, rigid and well-defined pore structures, thermal stabilities and tailorable surface charges with respect to other types of nanomaterials. Such properties of zeolites make them inevitable candidates for preparing ion-selective membranes for potentiometric cation sensing [4].

The hydrophilic character of zeolites also makes them very suitable materials for the co-immobilization of enzymes and mediators in the preparation of biosensors. The number and type of surface hydroxyl groups, which are important for immobilization applications, can be simply controlled by applying different heat treatment procedures. Finally, zeolites are known to be stable in both wet and dry conditions and well-tolerated by microorganisms, leading to an enhanced compatibility with biochemical analyses. All of these properties make zeolites unique nanomaterials and promising candidates for the immobilization of biological molecules and for advanced analytical tasks [5].

At present, some variants of biosensors containing zeolite crystals are known. As shown in [5], zeolites of various kinds can be effectively used for the glucose oxidase immobilization while developing glucose amperometric biosensors to optimize their sensitivity, selectivity and stability. As reported in [6,7], zeolites are used as alternative carriers for the enzyme immobilization while creating conductometric biosensors. Diverse variants of co-immobilization of urease and BEA-zeolites onto the surface of conductometric transducers were analyzed in respect to the improvement of analytical characteristics of biosensors for urea determination [8]. Promising results were obtained when zeolites were used for the development of urease biosensors based on pH-sensitive field-effect transistors [6,9-14].

There is also information on the benefits of application of zeolites as adsorbents for enzyme immobilization [15]. This option of immobilization allowed obtaining biomembranes on the surface of conductometric transducers without use of toxic substances.

In our work, we proposed a method of enzyme adsorption on zeolite monolayers for the creation of potentiometric biosensor. We have chosen the urease since it is one of the most studied and stable enzymes in biosensorics. In addition, the results obtained on urea biosensors can be applied to any other enzyme systems.

## Methods

### Materials

The materials used in this study were as follows: urease activity 66 U/mg, urea, glutaraldehyde (GA), glycerol and bovine serum albumin (BSA) (fraction V) from ‘Sigma-Aldrich Chemie’ (Munich, Germany). Silicalite, zeolite L, nano zeolite beta and calcine nano zeolite beta were synthesized in the Middle East Technical University (Ankara, Turkey). KH_2_P0_4_, NaOH and other substances used in the work were of domestic production and of chemical purity.

### Design of potentiometric transducer and measuring device

**Figure 1:**
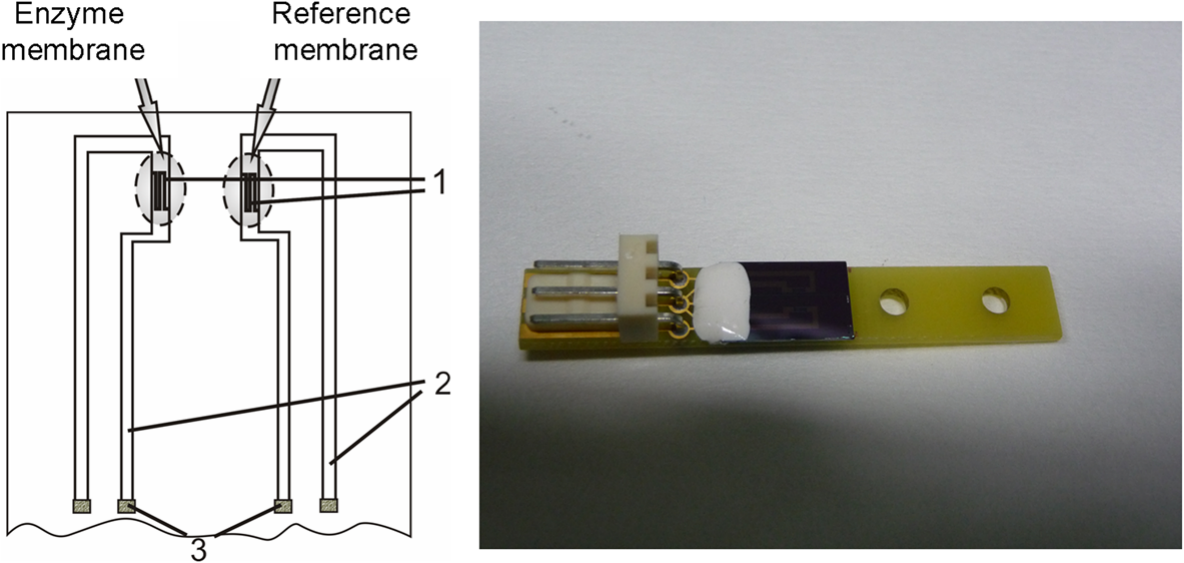
**Schematic and general views of components of ISFET elements.** 1, gate areas; 2, p ± diffusion buses from source and drain of each transistor; 3, aluminium contacts.

In the work, we used sensor chips with the differential pair of p-channel transistors on one crystal of total area 8 × 8 mm. The crystal included two identical transistors separated by 50-μm-wide protective n ± region with a contact to the substrate, the p + diffusion buses pulled to the chip edge with the contacts to the drain and source, and output to the integrated reference microelectrode. Schematic and general views of the sensor element are shown in Figure 1.

### Device for measurements

**Figure 2:**
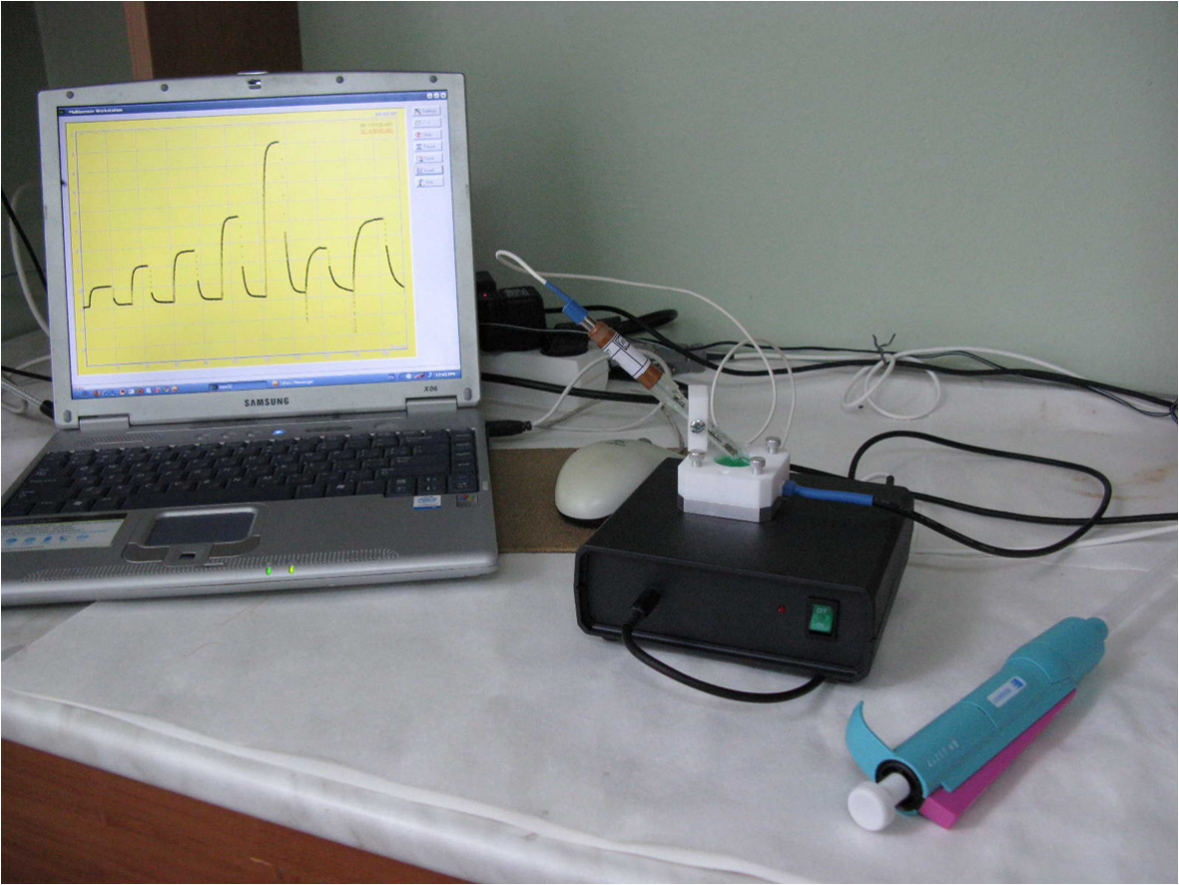
Portable device for measurement by ISFET connected to computer and measuring cell.

The experiments were performed on a digital portable device (Figure 2) with a circuit of direct measurement of the current in the channel of field-effect transistor with an active load. The output signal from the working cell with transducer enters the computer and is processed using «MSW_32» software. The device and sensor crystals were designed and manufactured at the V. Lashkaryov Institute of Semiconductor Physics, National Academy of Sciences of Ukraine.

### Immobilization of biological elements in glutaraldehyde vapour

To prepare the enzyme membrane, 5% urease solution was mixed with 5% BSA solution in 20 mM phosphate buffer (pH 7.4), which contained 10% glycerol (to stabilize the enzyme during immobilization and prevent early drying of the solution deposited on the transducer surface). To obtain the reference membrane, 10% BSA solution was prepared in 20 mM phosphate buffer containing 10% glycerol. A drop of the enzyme-BSA mixture was deposited on one fragment of the transducer-sensitive surface (selective membrane) and the BSA solution without enzyme on another (reference membrane). For membrane polymerization, the transducers were placed in atmosphere of saturated GA vapour for 20 to 25 min. The latter reacts with the available amino groups of proteins, contributing to the formation of cross links of Schiff base type (−N = CH−) in the membrane.

After immobilization, the transducers were dried in air and washed from GA excess in buffer solution for 10 to 15 min. Before measurements, the sensors with deposited biomaterial were kept for some minutes in the working buffer until a stable signal baseline is obtained.

### Procedure of zeolite synthesis

#### Silicalite

To synthesize the silicalite solution, we used 1TPAOH:4TEOS:350 × H_2_O. When hydrolysing tetraethoxysilane (TEOS) with tetrapropylammonium hydroxide (TPA-OH), we obtained a homogeneous solution by constant stirring for 6 h at room temperature. The crystallization took place at 125°C during 1 day. The material, which did not react, was removed from the solution by centrifugation. The size of silicalite particles was approximately 400 nm.

#### Nano beta zeolite

The molar composition of the nano beta zeolite is 0.25 Al_2_O_3_:25 SiO_2_:490 H_2_O:9 TEAOH. Silica source was TEOS (98%, Aldrich). Aluminium isopropoxide (98%, Aldrich), tetraethylammonium hydroxide (TEAOH) (20 wt.% in water, Aldrich) and double-distilled water were used as the other reactants. Ageing was continued under static conditions for 4 h with clear solution. The crystallization was completed within 17 days under static conditions at 100°C in Teflon-lined autoclaves. The product was purified by centrifugation [16]. Approximately, the particle size of nano beta zeolite is 60 nm.

#### Zeolite L

The molar composition of the nano zeolite L is 0.08Al_2_O_3_:1SiO_2_:0.5K_2_O:10H_2_O. Aluminium powder is dissolved in KOH solution. Colloidal silica (Ludox HS-40, Dupont, Wilmington, DE, USA) was then added under vigorous stirring, and the gel was stirred at room temperature for 5 min. The crystallization continued for 6 days in Teflon-lined autoclaves under static conditions at 170°C. Approximately, the particle size of nano zeolite L is 60 nm.

**Figure 3:**
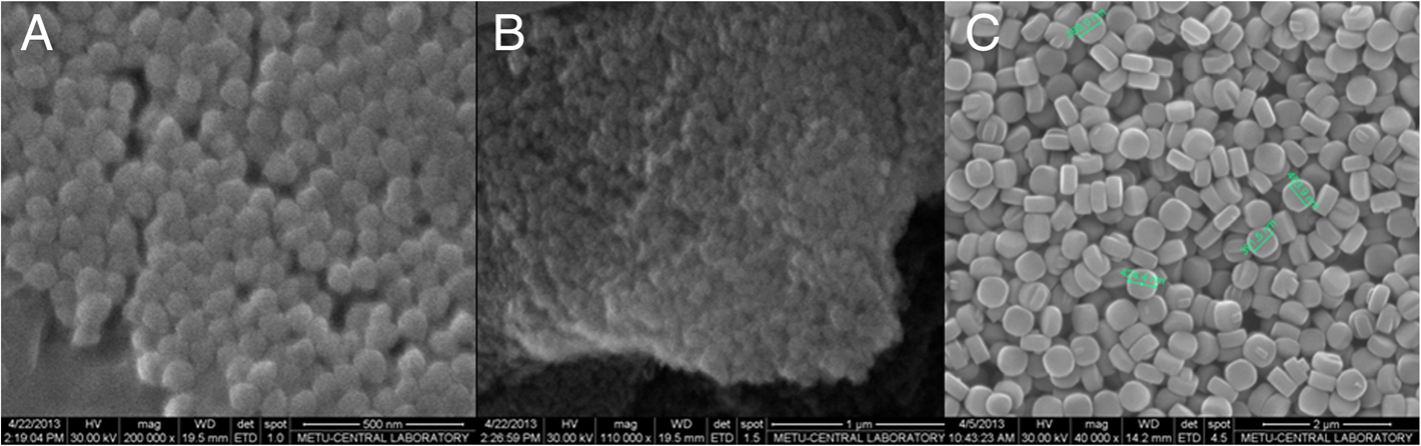
Scanning electron microscopy images of synthesized zeolites nano beta (A), nano zeolite L (B) and silicalite (C).

The results of scanning electron microscopy images of synthesized zeolites nano beta, nano zeolite L and silicalite are presented in Figure 3.

### Modification procedure for zeolite monolayers

Firstly, we tried to attach the zeolite on the electrodes surface but failed in our attempts. Then, we used a layer of poly(ethyleneimine) (PEI) between zeolite and electrode surface. In this case, zeolite attachment was attained but there was a homogeneity problem. To solve it, we used mucasol (1/6 *v*/*v*) in distilled water, which gave good results due to changing surface hydrophility.

The electrode surfaces were dip-coated with mucasol for 15 min, rinsed with copious amount of distilled water and dried under air. For the formation of homogeneous layers of PEI, both dip coating and spin coating techniques had been tried. Since spin coating gave more homogeneous layers, it was continued to be used. The effect of PEI solvent type (hot water and ethanol), PEI concentration (0.5%, 1%, 3%), spin coating time (3,000 rpm 15 s, 7 s) and calcination temperature (100°C, 90°C, 50°C) were investigated. The obtained monolayers were checked by microscope. The suitable conditions for zeolite monolayer production were chosen as follows: spin coating with 0.5% PEI in ethanol at 3,000 rpm during 15 s and calcination at temperature 90°C for 30 min.

The synthesized zeolites were directly attached to the obtained electrode surfaces simply by rubbing zeolites with a finger, a technique called direct attachment. These electrodes were used in further studies.

### Enzyme immobilization on the surface of zeolite monolayers

To produce the enzyme membrane, 5% urease solution in 20 mM phosphate buffer (pH 7.4) was prepared. To obtain the reference membrane, 5% BSA solution in 20 mM phosphate buffer was prepared. A drop of enzyme solution was deposited on one section of the transducer-sensitive surface, covered with zeolite, on another - BSA solution without enzyme (reference membrane).

After immobilization, the transducers were dried in air and washed from unbound components of membranes in buffer for 10 to 15 min. Before measuring, the sensors with deposited biomaterial were kept for some minutes in the working buffer until a stable signal baseline is obtained.

### Procedure of measurement by biosensor device

Measurements were conducted in 5 mM phosphate buffer, pH 7.4, at room temperature using a cell measuring system. The substrate concentration in the cell was specified by the addition of different aliquots of the substrate stock solutions to the working buffer. The experiments were performed in at least three repetitions. Nonspecific changes in the output signal because of fluctuations in temperature, pH and electrical breakthrough were significantly reduced due to the differential mode of measurements.

## Results and discussion

The aim of our work was to improve analytic characteristics of the enzyme-based biosensors. We have chosen the urease, since it is one of the most studied and stable enzymes in biosensorics. We studied the method of urease adsorption on the surface of pH-sensitive field-effect transistors (ISFET) using monolayer of different types of zeolites: silicalite, nano beta zeolite, zeolite nano L and calcinated nano beta zeolite. Obtained results were compared with results of biosensors based on urease immobilized in GA vapour.

The functioning of enzyme urease biosensor is based on the reaction of urea cleavage to ions NH_4_^+^ with the consumption of protons:

This reaction results in the change of pH inside selective membrane which is recorded by pH-sensitive field-effect transistors.

**Figure 4:**
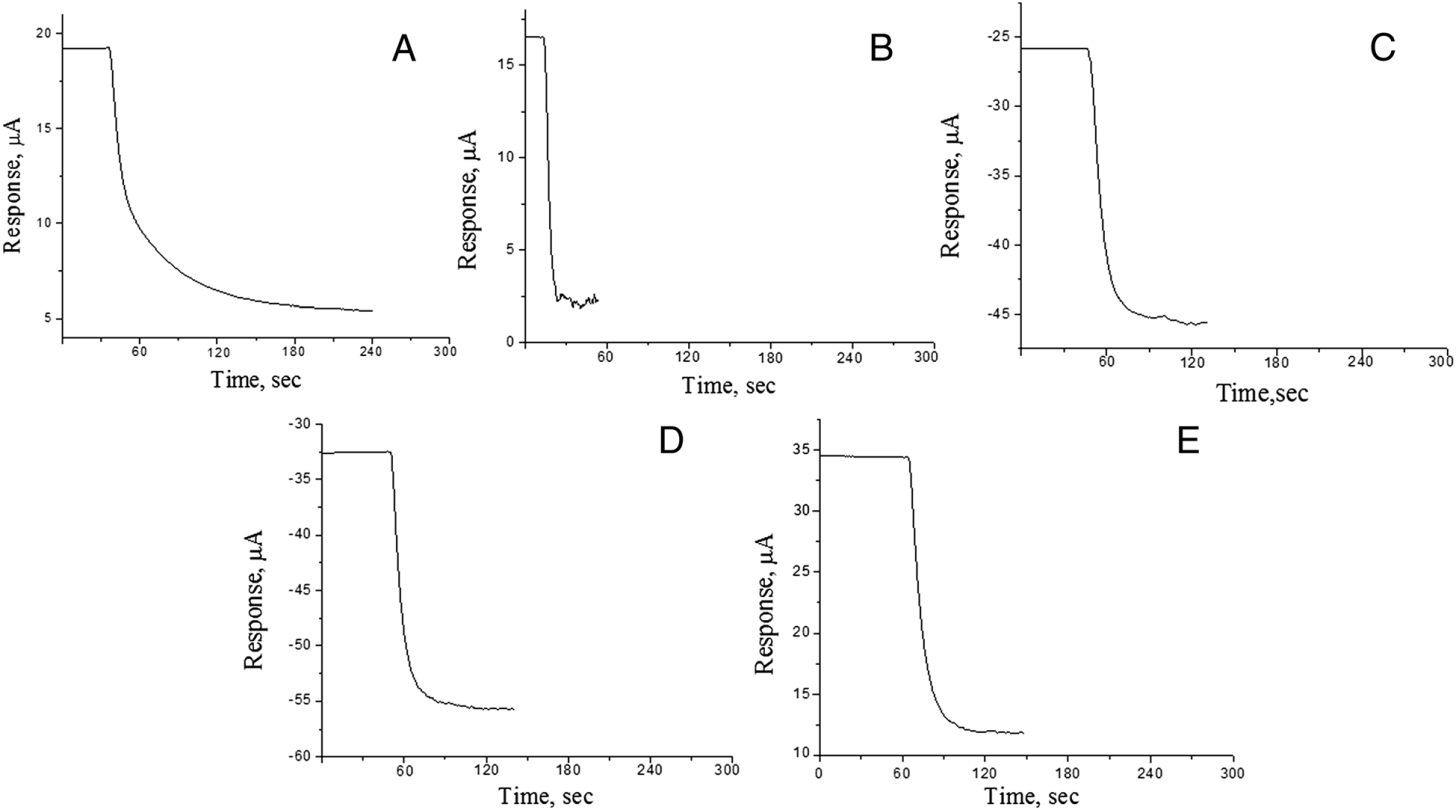
**The biosensor responses to the same concentration of urea for different types of immobilization.** Typical responses to 0.5 mM urea obtained with biosensors created by urease immobilization in GA vapour **(A)**, adsorption on the surface of transducer covered with: monolayer of silicalite **(B)**, monolayer of nano beta zeolite **(C)**, monolayer of zeolite L **(D)**, monolayer of calcinated nano beta zeolite **(E)**. The measurements were carried out in 5 mM phosphate buffer solution (pH 7.4).

First of all, to test the efficiency of the proposed method of enzyme immobilization, it was necessary to compare typical responses of the biosensors obtained by the traditional method of immobilization and those with the immobilization by adsorption. Therefore, we measured the biosensor responses to the same concentration of urea (0.5 mM) for the following types of immobilization: in GA vapour (Figure 4A), adsorption on the surface of monolayers of silicalite (Figure 4B), nano zeolite beta (Figure 4C), zeolite L (Figure 4D) and calcined nano zeolite beta (Figure 4E). As seen, the biosensors with zeolite-immobilized urease demonstrated the response time to urea about 1 min, which is three times less compared with that for the biosensors based on urease immobilized in GA vapour. Thus, the proposed method of urease adsorption on zeolites can significantly reduce the time of biosensor analysis.

**Figure 5:**
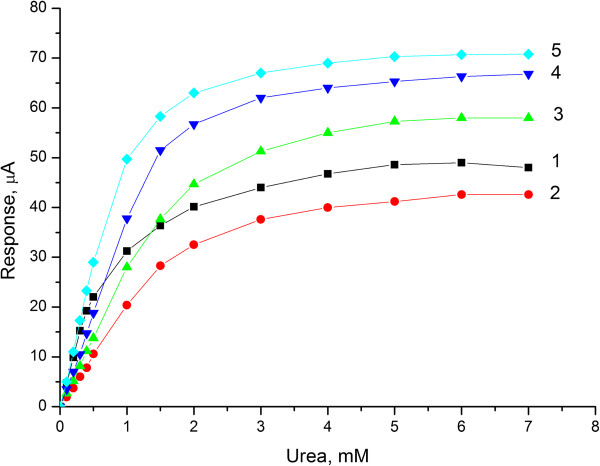
**Calibration curves for urea determination obtained for biosensors created by urease immobilization.** In GA vapour (1), adsorption on transducers covered with monolayer of: silicalite (2), nano beta zeolite (3), zeolite L (4), calcinated nano beta zeolite (5). The measurements were carried out in 5 mM phosphate buffer solution (pH 7.4).

Important working characteristics of the biosensors for analysis of substances in real samples are their sensitivity and linear range of determination. Therefore, we compared calibration curves of the biosensors based on different types of immobilization. For this, urea of various concentrations ranging from 0.1 mM up to saturation was added to the measuring cell. The obtained calibration curves (Figure 5) show that the highest sensitivity has the biosensors based on urease immobilized on the surface of monolayer of calcined nano zeolite beta. The biosensors with urease adsorbed on monolayers of silicalite and nano zeolite L have the largest linear range while the smallest was revealed for the biosensor with urease immobilized in GA vapour.

**Figure 6:**
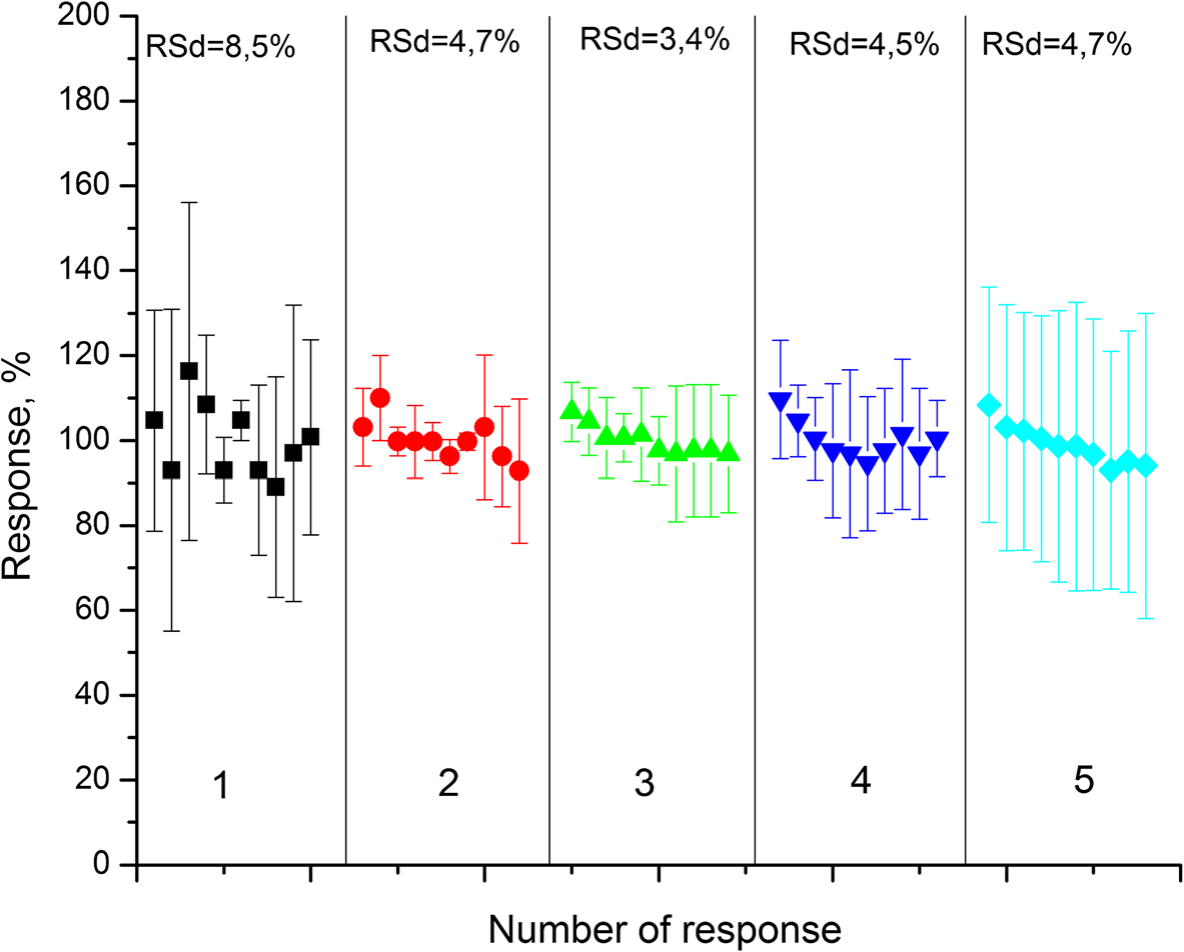
**Reproducibility of responses to the 0.5 mM urea obtained with biosensors created by different methods of urease immobilization.** In GA vapour (1), adsorption on the surface of transducer covered with monolayer of: silicalite (2), nano beta zeolite (3), zeolite L (4) and calcinated nano beta zeolite (5). The measurements were carried out in 5 mM phosphate buffer solution (pH 7.4).

At the next stage, we investigated the reproducibility and inter-reproducibility of the created biosensors, as these characteristics determine their stability. It was necessary to verify whether the new method of immobilization along with an increase in sensitivity and linear range provides good response reproducibility. First of all, we examined reproducibility of the biosensor responses to the same urea concentration (0.5 mM). We added the corresponding aliquot of concentrated urea solution to the cell, obtained the response, washed the sensor from the reaction products in working buffer and repeated the measurements 10 times. As 100% was taken, the mean value calculated for all the responses was obtained for certain type of immobilization. For each type of immobilization, the experiment was repeated at least three times. The results are presented in Figure 6.

As seen, the best signal reproducibility manifested the sensor obtained by immobilization on a monolayer of nano zeolite beta; the worst was obtained for immobilization in GA vapour. Thus, the conclusion can be made that immobilization by deposition of selective membrane on the zeolite-modified surface results in noticeable improvement of reproducibility and thus to the enzyme stabilization.

**Figure 7:**
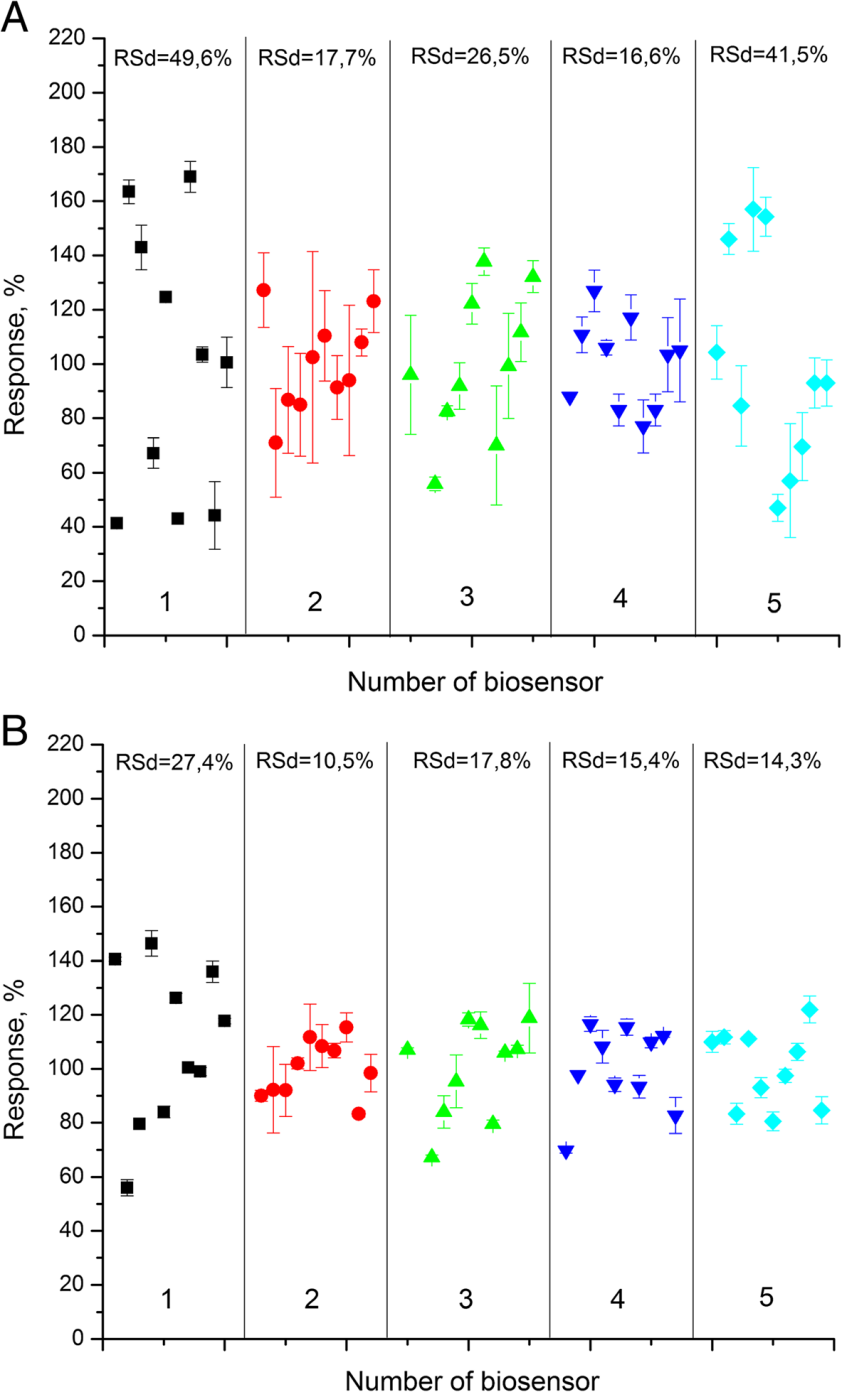
**Inter-reproducibility of signals to urea concentration.** 0.5 mM **(A)** and 3.5 mM **(B)** obtained with biosensors created by urease immobilization in GA vapour (1), adsorption on the surface of transducer covered with monolayer of: silicalite (2), nano beta zeolite (3), zeolite L (4), calcinated nano beta zeolite (5). The measurements were carried out in 5 mM phosphate buffer solution (pH 7.4).

Inter-reproducibility is a reproducibility of the responses of a number of biosensors to the same substrate concentration under the enzyme immobilization on different transducers. We selected 10 ISFETs with similar working characteristics. The experiment was conducted on biosensors with different methods of immobilization. We deposited a selective membrane on the surfaces of all transducers and then measured and compared the responses to addition of the substrate of the same concentration. Two concentrations were taken: 0.5 mM, which corresponds to the biosensor linear range, and 3.5 mM which is a saturation value. After receiving the responses, the sensor was completely washed from enzyme and reference membranes, as well as from zeolite on its surface. Next, new immobilization was carried out and responses to the same substrate concentration were measured. The described procedure was repeated 10 times. For each sensor, the responses to certain concentration were obtained in three repetitions, and the average value was calculated (Figure 7). As 100% was taken, a mean value calculated for all the responses was obtained upon corresponding type of immobilization.

As seen, the slightest data dispersion (i.e. the best inter-reproducibility) at both urea concentrations was revealed for the biosensor obtained by urease immobilization on the surface of monolayer of silicalite and zeolite L. The inter-reproducibility for the biosensors immobilized by adsorption on the surface of monolayers of nano zeolite beta and nano calcined zeolite beta was almost the same; the worst was observed for biosensors based on urease immobilized in GA vapour.

The comparative data (noise, baseline drift, minimum detection limit, linear range, error of signal reproducibility) for the biosensors based on different types of immobilization are shown in Table 1.

**Table 1:** Comparison of operational characteristics of biosensors created using different types of urease immobilization

**Type of immobilization**	**Noise (μA)**	**Drift of base line (μA/min)**	**Detection limit (mM)**	**RSd of reproducibility of signal (%)**	**RSd of inter-reproducibility of immobilization (%)**	**Linear range (mM)**
In GA vapour	0.02	0.010	0.0015	8.5	27.4	0 to 0.5
On the surface of silicalite monolayer	0.03	0.028	0.0015	4.7	10.5	0 to 1.5
On the surface of nano beta zeolite monolayer	0.03	0.001	0.0015	3.4	17.8	0 to 1.0
On the surface of zeolite L monolayer	0.06	0.004	0.0050	4.5	15.4	0 to 1.5
On the surface of calcinated nano beta zeolite monolayer	0.09	0.013	0.0055	4.7	14.3	0 to 1.0

The obtained data show that adsorption on zeolites can promote a linear range, a decrease in the error of response reproducibility and in baseline drift. Nevertheless, the biosensors obtained by traditional immobilization in GA vapour can be characterized by lower baseline noise.

Thus, the technique of enzyme adsorption on zeolite monolayers allows us, on the one hand, to avoid using toxic substances (glutaraldehyde, etc.), on the other - to obtain potentiometric biosensors with improved analytical characteristics.

## Conclusions

We investigated a possibility of application of a new method of immobilization of enzyme urease on the surface of pH-sensitive field-effect transistors by adsorption on zeolite monolayers. In the experiment, we used different types of zeolites: nano beta, calcinated nano beta, silicalite and nano zeolite L. As a control, we used the method of immobilization in glutaraldehyde vapour (without zeolite). It is shown that the enzyme immobilization on monolayers of silicalite or nano zeolite L results in increasing biosensor linear range up to 1.5 mM. We found that the biosensors based on adsorbed urease were characterized by better signal reproducibility during work and better reproducible stable immobilization of the biological material. The biosensors obtained with new methods were compared by various parameters: noise, baseline drift, minimum determination limit, error of signal reproducibility, error of immobilization reproducibility and width of the linear range of operation.

It was found that the use of monolayers of different zeolites as a carrier for adsorption of the enzyme for the creation of potentiometric biosensors can result in increasing linear range of their operation, reducing the minimum limit of urea determination, improved response reproducibility and inter-reproducibility and decreasing time of analysis.

## Competing interests

The authors declare that they have no competing interests.

## Authors’ contributions

MKS performed the experiments to study the effect of different methods of immobilization on the biosensors operation and drafted the manuscript. OOS planned and supervised the experiments performed by MKS and wrote and arranged the article. VMA monitored the performance of measuring devices and tuned the transistors. BOK was involved in the synthesis of zeolites and silicalite and took part in the deposition of silicalite onto the transistors surface. BA proposed an idea of using zeolites for enzymes adsorption and controlled the zeolites synthesis by electron microscopy. SVD is a supervisor of the whole work, the results of which are presented in this article and he proposed an idea of the development of potentiometric biosensors based on enzyme adsorption on zeolites. All authors read and approved the final manuscript.
